# ST-Segment Elevation Myocardial Infarction and Normal Coronary Arteries after Consuming Energy Drinks

**DOI:** 10.1155/2017/4061205

**Published:** 2017-01-19

**Authors:** S. Michael Gharacholou, Nkechinyere Ijioma, Emma Banwart, Freddy Del Carpio Munoz

**Affiliations:** ^1^Division of Cardiology, Mayo Clinic Health System-Franciscan Healthcare, La Crosse, WI, USA; ^2^Division of Cardiology, Mayo Clinic, Rochester, MN, USA

## Abstract

The use of energy drinks, which often contain stimulants, is common among young persons, yet there have been few reports of adverse cardiac events. We report the case of a 27-year-old man who was admitted to our facility with an acute ST-segment elevation myocardial infarction in the setting of using energy drinks. Angiography revealed no obstructive coronary disease. The patient had elevation of cardiac troponin. Noninvasive testing with echocardiography and cardiac magnetic resonance imaging demonstrated both abnormalities in resting wall motion at the anterior apex along with late gadolinium enhancement of the anterior wall, respectively. The patient also underwent formal invasive evaluation with an intracoronary Doppler study demonstrating normal coronary flow reserve and acetylcholine provocation that excluded endothelial dysfunction and microvascular disease. The patient recovered and has abstained from consuming additional energy drinks with no reoccurrence of symptoms. A review of some of the potential cardiac risks associated with consuming energy drinks is presented.

## 1. Case Report

**Figure 1:**
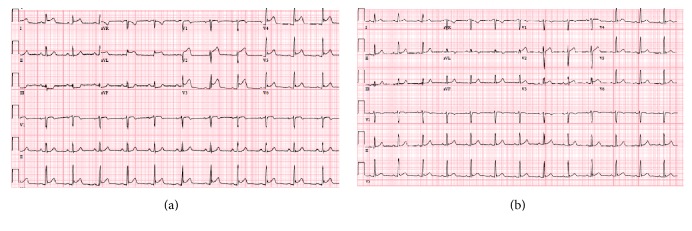
Acute anterolateral ST-segment elevation myocardial infarction (a). Note ST elevations in anterior precordial leads V2–V5, lateral limb leads I and aVL, and reciprocal changes in III and aVF. Resolution of ST-segment abnormalities and inferior reciprocal changes after sublingual nitroglycerin and intra-arterial nitroglycerin and verapamil at the time of angiography via radial sheath (b).

**Figure 2:**
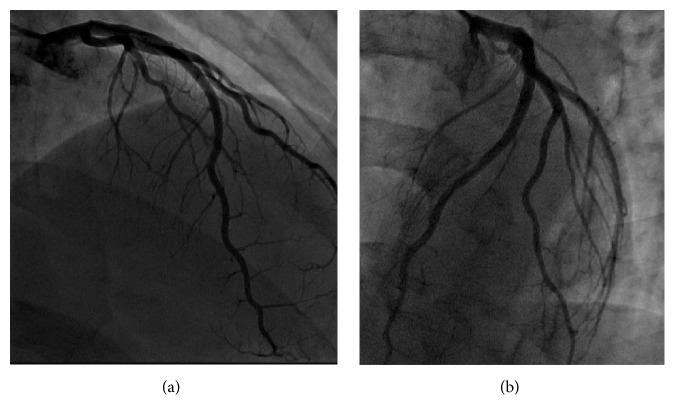
Straight AP cranial (a) and left anterior oblique cranial (b) projections of normal left anterior descending coronary artery.

**Figure 3:**
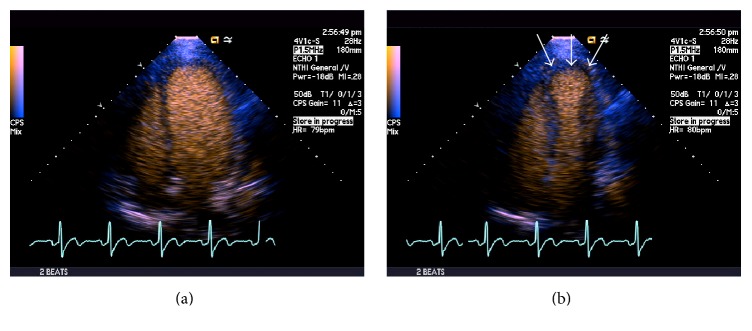
Apical four-chamber echocardiographic images with contrast enhancement at end-diastole (a) and end-systole (b) showing severe akinesis of the left ventricular apex (arrows).

**Figure 4:**
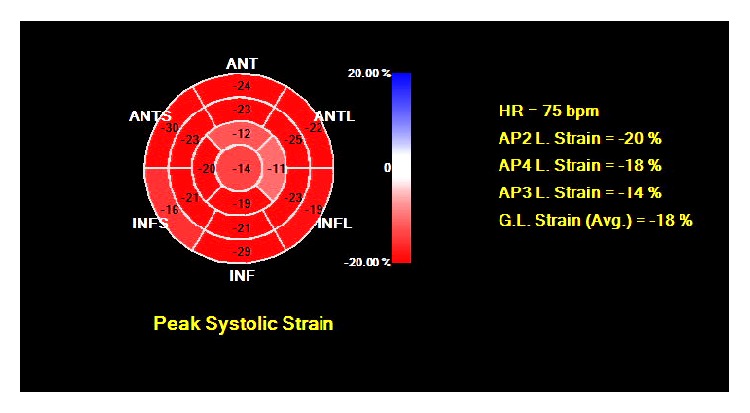
Abnormal longitudinal strain function confined to the left ventricular apex.

**Figure 5:**
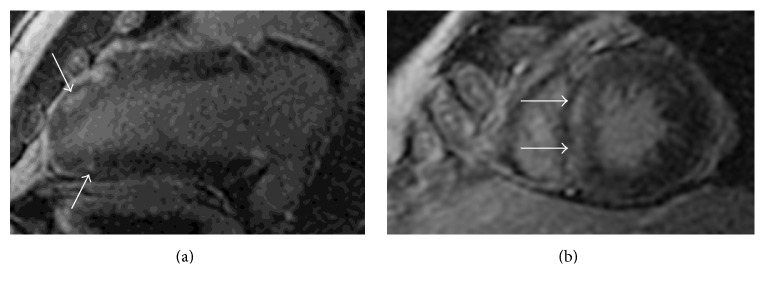
Cardiac magnetic resonance imaging with demonstration of patchy and some full-thickness late gadolinium enhancement of the (a) distal anteroapical wall segments, inferoapical segment, and (b) mid septum (arrows).

A 27-year-old man with no previous medical history presented to the emergency department with 1 hour of acute onset of left-sided chest pain, shortness of breath, and diaphoresis. He reported active tobacco smoking but denied other drug use. He used nonsteroidal anti-inflammatory medications for back pain. There was a family history of coronary artery disease in an uncle who underwent coronary revascularization. He had also recently been using energy drinks (Rockstar, Rockstar, Inc., Las Vegas, NV), sometimes 4-5 beverages in a 12-hour period, in order to stay awake during his evening work shift in a warehouse. He denied any previous episodes of chest pain, exertional symptoms, previous thromboembolic disease, nausea or vomiting, or syncope. On examination, his blood pressure was elevated at 155/100, pulse was 74 beats per minute, respiratory rate was 18 breaths per minute, and oxygen saturation was 100% on 2 liters of supplemental oxygen. His cardiac examination was normal, including a normal first and second heart sound and no appreciable murmur or pericardial friction rub. His laboratory values were notable for hemoglobin of 15.4 g/dL, white blood cell count of 5.8, platelet count 254,000, normal blood chemistry, and an elevated troponin T of 0.41 (normal reference range < 0.01 ng/mL) and CK-MB of 123.4 (normal reference range < 7.8 ng/mL). His electrocardiogram demonstrated ST-segment elevations in leads V2–V6, I, and aVL along with reciprocal changes in leads III and aVF, concerning for an acute anterior ST-segment elevation myocardial infarction (STEMI) (Figure 1(a)). He was treated with sublingual nitroglycerin, unfractionated heparin, and aspirin in the emergency department which improved but did not completely alleviate his symptoms and brought urgently to the cardiac catheterization laboratory. Coronary angiography was performed from a radial approach and symptom relief was documented after intra-arterial injection of a radial antispasmodic cocktail (i.e., 200 mcg nitroglycerin, 2.5 mg verapamil, and 5,000 units of heparin). Coronary angiography showed normal coronary arteries with TIMI III flow without any angiographic evidence of stenosis, dissection, embolism, or plaque rupture (Figure 2). A repeat electrocardiogram after his coronary angiogram demonstrated normalization of his ST-segment abnormalities (Figure 1(b)). An echocardiogram was performed and demonstrated significant akinesis of the left ventricular apex (Figures 3(a) and 3(b)), findings that were confirmed with abnormal longitudinal strain in the apical segments (Figure 4). Cardiac magnetic resonance imaging (cMRI) also documented akinesis in the left ventricular apex, similar to the wall motion abnormalities present on echocardiography. In addition, cMRI confirmed areas of abnormal delayed gadolinium enhancement in both a patchy and full-thickness configuration along the mid and distal anterior wall and the mid septum (Figures 5(a) and 5(b)). A formal toxicology examination was not requested given that the patient rapidly recovered after his initial presentation. The absence of the toxicology leaves the question as to whether other potential substances could have been associated with the acute cardiac presentation. A urine drug screen at the time of admission was negative for cocaine, amphetamines, and barbiturates. The patient was treated with long acting nitrates and calcium channel blocker, though he continued to have occasional episodes of chest discomfort. Given the concern of recurrent spasm and given that the presumed etiology of his ST-elevation myocardial infarction was coronary vasospasm, he was referred for repeat coronary angiography, including a functional protocol to test the response of the coronary endothelium and the coronary microcirculation. His coronary angiogram was normal, with no evidence of atherosclerosis or vasospasm. Intracoronary adenosine was administered at a dose of 60 mcg and coronary flow reserve (CFR) was normal at 3.0 using intracoronary Doppler study in the left anterior descending (LAD) artery. These findings suggested normal microcirculatory function. Subsequently, increasing concentrations of intracoronary acetylcholine were administered with no significant change in the Doppler-derived average peak velocity, CFR, symptoms, angiographic appearance of the LAD, or electrocardiographic parameters, all consistent with normal endothelial function. Based on these results, he was not felt to have any functional coronary or endothelial abnormalities. He was advised to abstain from any further use of energy drinks.

## 2. Discussion

We report a case of anterior STEMI in the absence of coronary obstruction presumably due to severe coronary vasospasm and temporally related to drinking commercially available caffeinated energy drinks. To our knowledge, our report represents the second report in the literature of energy drinks precipitating an episode of STEMI that also underwent invasive angiography with no evidence for coronary obstruction [1] and the only report that evaluated the pattern of myocardial abnormality of energy drink-induced STEMI using noninvasive imaging modalities of 2-dimensional echocardiography, longitudinal strain, and cMRI.

Up to 30% of women and 12% of men have myocardial infarction but no evidence of obstructive (≥50%) coronary disease at the time of angiography [2, 3]. From a mechanistic standpoint, these cases have been attributed to plaque disruption that cannot be reliably detected by invasive angiography, endothelial and microvascular dysfunction, or vasospasm [4]. In one study of patients with myocardial infarction and nonobstructive CAD, nearly 60% had abnormal cMRI findings in a transmural or subendocardial pattern (i.e., ischemic) [3]. Interestingly, the remaining 40% had subepicardial or midwall (i.e., nonischemic) or mixed (both ischemic and nonischemic) patterns of late gadolinium enhancement on cMRI [3]. The proposed mechanism for this might be related to focal ischemia in an area of vasospasm or plaque erosion, resulting in platelet aggregates and distal embolization [3]. This ischemic insult can be appreciated with T2 hyperintensity on cMRI, as myocardial edema is often a consequence of myocardial ischemia. Coronary vasospasm has been implicated as a cause of myocardial infarction when no obstructive CAD is identified at the time of coronary angiography, and certain clinical presentations, such as cocaine and stimulant use, are risk factors for development of coronary vasospasm. Tobacco use has been implicated in coronary spasm, likely mediated through nicotine, which can be a potent vasoconstrictor.

Energy drinks were introduced in the US in 1997, approximately 10 years after first being released in Europe. Their popularity among young adults has steadily increased and it is estimated that about 1 out of 20 young men consume a daily energy drink, with sales of energy drinks in the US exceeding $9 billion [5]. Because many of these products contain herbal or “natural” ingredients, they avoid federal regulation that is required of medications, despite the increasing concern regarding adverse health effect and unexpected deaths among users of energy drinks. The Food and Drug Administration has previously declared energy drinks containing both caffeine and alcohol as being unsafe. No restrictions apply to how energy beverage companies advertise “energy” or “performance” effects of their product [6]. Finally, the caffeine content in energy beverages are often not reported and, when reported, may vary widely. Caffeine absorbs quickly after ingestion and its liver metabolites theophylline and theobromine are active stimulants. Caffeine overdoses, some fatal, have been reported and purportedly attributed to cardiac arrhythmia [7]. Energy drinks are increasingly becoming a public health problem as more individuals are seeking stamina and alertness, yet the safety of these beverages and potential drug-drug interactions that may occur have not been well described.

Svatikova et al. performed a randomized, placebo-controlled, crossover trial of 25 healthy volunteers who consumed 16 oz of a commercially available energy drink or placebo (matched in taste and color but without caffeine or stimulants) and studied effects on blood pressure, heart rate, and serum norepinephrine levels [8]. Despite no differences in heart rate between energy drink use and placebo, there was a significant increase in systolic, diastolic, and mean blood pressure with energy drink use as compared to placebo. This was associated with a significant increase in norepinephrine levels after energy drink (149.8 pg/mL to 249.8 pg/mL) as compared to placebo (139.9 pg/mL to 178.6 pg/mL) (*p* = 0.003). These findings suggest that adrenergic stimulation with energy drinks may predispose patients to cardiovascular risk and may be particularly concerning in patients with unrecognized cardiac disorders or cardiac-related susceptibilities. A recent systematic review of published cases of adverse cardiovascular events after consuming energy drinks identified only 4 cases of STEMI, which occurred in men between the ages of 17 and 24 [9]. Two of the cases never underwent coronary angiography. Of the 2 cases that underwent cardiac catheterization, 1 patient had intracoronary thrombosis of the left main coronary artery and required emergency surgical coronary bypass [10]. The other case had no obstructive coronary disease, which was similar to ours; however, that patient had a normal echocardiogram with no wall motion abnormalities [1].

Our case adds to the current literature on STEMI precipitated by energy drink consumption in a patient with angiographically normal coronary arteries, representing the second such case reported. In addition, unique aspects of this report include cardiac structural findings documenting regional wall motion abnormalities by 2-dimensional echocardiography, longitudinal strain, and cMRI. Tissue characterization by cMRI demonstrated abnormal late gadolinum enhancement in the myocardial territory supplied by the left anterior descending artery, a pattern that previously has been reported in patients suffering acute myocardial infarction but without obstructive coronary disease. Longitudinal strain and cMRI findings of energy drink-induced STEMI have not previously been reported. Given that the patient reported a smoking history, tobacco use represents a potential confounder regarding his clinical presentation with STEMI. Although there were no angiographic findings to suggest the presence of atherosclerosis, as would often be seen in chronic smokers, nicotine products have been implicated in cases of vasospasm. Clinicians should be aware of the potential cardiovascular adverse events of energy drinks in young persons who present with acute cardiovascular issues, particularly given the growing use of these energy beverages in both US and world markets.
